# The roles of scene priming and location priming in object-scene consistency effects

**DOI:** 10.3389/fpsyg.2014.00520

**Published:** 2014-05-30

**Authors:** Nils Heise, Ulrich Ansorge

**Affiliations:** Faculty of Psychology, Institut für Psychologische Grundlagenforschung und Forschungsmethoden, Universität WienWien, Austria

**Keywords:** visual search, attention, priming, contextual cuing, consistency priming

## Abstract

Presenting consistent objects in scenes facilitates object recognition as compared to inconsistent objects. Yet the mechanisms by which scenes influence object recognition are still not understood. According to one theory, consistent scenes facilitate visual search for objects at expected places. Here, we investigated two predictions following from this theory: If visual search is responsible for consistency effects, consistency effects could be weaker (1) with better-primed than less-primed object locations, and (2) with less-primed than better-primed scenes. In Experiments 1 and 2, locations of objects were varied within a scene to a different degree (one, two, or four possible locations). In addition, object-scene consistency was studied as a function of progressive numbers of repetitions of the backgrounds. Because repeating locations and backgrounds could facilitate visual search for objects, these repetitions might alter the object-scene consistency effect by lowering of location uncertainty. Although we find evidence for a significant consistency effect, we find no clear support for impacts of scene priming or location priming on the size of the consistency effect. Additionally, we find evidence that the consistency effect is dependent on the eccentricity of the target objects. These results point to only small influences of priming to object-scene consistency effects but all-in-all the findings can be reconciled with a visual-search explanation of the consistency effect.

## Introduction

Semantic knowledge from long-term memory (LTM) allows humans to search for a picture among distractor pictures in the glimpse of an eye: Potter (1975, 1976) has shown that participants were able to detect a visual target image among distractor images where even an incidental short-term memory of the pictures was prevented. Such LTM representations can also concern the probabilities of certain objects' appearances in specific contexts (Biederman et al., 1973; Bar, 2004, 2009). Supportive evidence for this claim comes in the form of a consistency effect: perceiving a scenic context can facilitate or impede the recognition of objects depending on the semantic fitting between scenes and objects (Biederman et al., 1973, 1982; Biederman, 1995; Bar, 2004; Davenport and Potter, 2004; Davenport, 2007; Mudrik et al., 2011; see also, e.g., Hollingworth and Henderson, 1998, 1999; Henderson and Hollingworth, 1999). Biederman et al. (1982), for instance, presented objects embedded in familiar or unfamiliar scene contexts. They found that the participants were better at reporting scene-consistent than inconsistent objects. For example, if the scene depicted a kitchen, participants were faster at recognizing a frying-pan than a hydrant. Thus, observers seemingly drew on LTM representations about the probabilities of certain objects' appearances in specific contexts. This result was confirmed by other studies using alternative testing methods to investigate this consistency effect (Antes et al., 1981; Boyce et al., 1989; De Graef et al., 1990; Boyce and Pollatsek, 1992; Ganis and Kutas, 2003; Davenport and Potter, 2004; Davenport, 2007).

However, the consistency effect might additionally be modulated by *visual priming* once (1) objects are presented at varying positions within the scene images; and (2) either images, objects, or their locations are repeated. Once objects are presented at varying positions, correct object recognition also depends on a successful “visual search” for an object (e.g., Wolfe et al., 2011)^1^. This is illustrated by research of Torralba et al. (2006). These authors presented their participants with photographs of natural scenes under varying search instructions. It turned out that the participants' fixation directions were heavily influenced by the specific search instructions. When the participants searched for people, they directed their fixations preferentially to the ground level within the images. When the participants searched for mugs or pictures, however, with the same photographs the participants' gaze was primarily directed toward the surfaces of tables and walls, respectively. These findings show that under conditions of varying object locations consistent objects can be found more efficiently than inconsistent objects because the gaze is primarily directed toward the most likely locations of searched-for objects.

Importantly, once participants have to search through the images, visual priming is possible. Usually, priming effects consist in facilitated performance for repeated as compared to non-repeated visual inputs. For instance, Maljkovic and Nakayama (1996) presented their participants with to-be-searched-for objects at varying positions. Across consecutive trials, the objects were presented at the same position or at switched positions. A clear position priming effect was found: searching for objects was faster at repeated than switched positions. Although many typical priming effects are studied over relatively short time scales (e.g., from one trial to the next trial), priming is also found across substantially larger time spans (of several trials, see Maljkovic and Martini, 2005, and up to months, see Cave, 1997). Here, we specifically studied whether two well-known priming influences in visual search may affect the size of the consistency effect. The priming influences that we studied were: (1) contextual cueing—the facilitation of visual search for objects at repeated vs. non-repeated locations where scene contexts repeat together with the object positions (Chun and Jiang, 1998, 1999)^2^; and (2) scene priming—the facilitation of scene recognition for similar or repeated scenes (Xu et al., 2007; Melcher, 2010)^3^.

Critically, contextual cueing could help locating both, consistent, and objects. This could diminish or boost the consistency effect. In particular, assuming that only consistent objects can already be recognized efficiently on the basis of scenes that are associated with the objects in LTM (Bar, 2004; Davenport and Potter, 2004), factors such as location priming and contextual cueing could especially benefit the search for scene-inconsistent objects more than that of consistent objects. Thus, theoretically, location priming has the potential to diminish the object-recognition differences between consistent and inconsistent conditions.

In addition, facilitated processing of primed scenes could boost the object-scene consistency effect, given that scene information of repeated scenes can be registered better and, hence, could have a higher influence on searching for objects. To test the influences of scene repetitions, location repetitions, and their combined effects in Experiments 1 to 2, we used B/W outline images and presented the objects at changing locations within repeated scenes.

## Experiment 1

So far, consistency effects were assessed with data averaged across all repetitions of scenes, and the possible number of object locations within one specific scene was not systematically varied. Therefore, little attention has been devoted to studying how contextual cueing or scene priming might change the consistency effect during the course of an experiment.

Experiment 1 closes this gap. To test whether consistency effects are affected by contextual cueing within a particular scene (Hollingworth, 2006, 2007), we varied the number of positions that an object could occupy within a scene. During repeated presentations of scene backgrounds, objects were always presented at the same position, while in a second condition objects were presented at one out of two, or in a third condition at one out of four different positions within a particular scene context.

If the certainty about the position of an object helps finding the object, we expected an improvement of object recognition performance that is inversely proportional to the number of locations an object could occupy in a scene. Also, the same prediction can be made on the basis of contextual cueing because the repetitions of object locations were at the same time repetitions of the objects in a particular scene.

Importantly, contextual cueing could also modify the consistency effect. If an inconsistent object, for which so far there is less support by LTM-associated scene recognition, is repeatedly presented at the same position, this might facilitate finding and recognizing of this object. This facilitation for an inconsistent object could well exceed the helpful influence of contextual cueing on the recognition of consistent objects because recognizing these objects could benefit from associated scene contexts right from the beginning of the experiment, leaving less space for an improvement by contextual cueing across trials. In other words, contextual cueing is a factor potentially reducing the disadvantage on object recognition for inconsistent objects.

In addition, regardless of contextual cueing, scene recognition might benefit from scene repetitions (Xu et al., 2007). Across the current experiment, each scene was repeated several times. This means that scene priming could boost scene recognition as a function of time on task or number of scene repetitions. Because scene recognition would generally allow for a more frequent head start of scene recognition over object recognition, scene priming has the potential to increase the differential effect of scenes on the search for consistent and inconsistent objects and, thus, to boost the consistency effect.

### Methods

#### Participants

Forty-five students (mean age 22 years) participated in the experiment in exchange for 5 € or course credit. All of them reported having understood the instructions and the meaning of the target words, had normal or corrected-to-normal vision, and were naïve with respect to the hypotheses.

#### Apparatus

A computer with a 17-inch TFT color monitor (resolution 1024 × 768 pixels; refresh rate 60 Hz) registered reaction times (RTs) given via a standard keyboard. The experiment was run using E-Prime software (Version 1).

#### Stimuli and procedure

**Figure 1:**
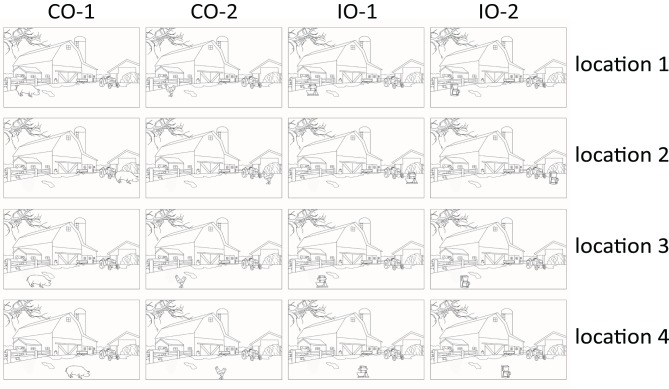
**Illustration of stimuli used in Experiments 1 and 2**. Depicted is one scene, and how it was presented with objects in different locations (in different rows). Each scene was shown with two objects of the consistent type (CO-1 and CO-2, 1st and 2nd column) and two of the inconsistent type (IO-1 and IO-2, 1st and 2nd columns). The scene represents a farm, with the objects in the lower left corner of the image. Consistent objects are a pig and a chicken; inconsistent objects are a mixer and a coffee machine. (Note that the identity of consistent and inconsistent objects was fully balanced across the images. A mixer and a coffee machine, for example, were used as scene-consistent objects in kitchen images. This is not depicted.) The words *Starten*, *Huhn*, and *Schwein* are German for *start*, *chicken*, and *pig*, respectively.

Stimuli were black (5.6 cd/m^2^) on white (147.0 cd/m^2^) and were presented in a viewing distance of 70 cm. We combined 22 different scene contexts × 2 consistent objects × 2 inconsistent objects × 4 locations. Specifically, we cut out the objects from the original pictures used by Hollingworth and Henderson (1998) and inserted them using the GIMP open source software package (Gnu Image Manipulation Program, http://www.gimp.org) on ecologically plausible positions in the scenes. Half of the resulting 352 pictures were consistent (e.g., a harp as an object on the stage of a concert hall as a scene) and half were inconsistent (e.g., a motorbike as an object presented on the same stage)^4^. Table A.1 in the Supplementary Material provides a list of the objects and scenes that we used. Scenes subtended a visual angle of 28.5° (width) × 21.5° (height) and objects varied in side length from 0.7° to 6.1°. Each participant saw all 22 scene backgrounds with each of its four possible objects two times (i.e., with correct answer labels displayed once to the right, once to the left; see Figure 1) resulting in eight repetitions of each scene. Two of the four objects were consistent with a scene and two were inconsistent. Also, across images, each object was equally often consistent and inconsistent to a scene.

Also, the number of locations varied independently of the object identities. The number of locations varied between participants, with objects presented either at only one location, at two alternative locations, or at four alternative locations within a specific scene. All selected locations were plausible—that is, all objects were presented where a depicted surface would support the objects. This was done because physically impossible locations (e.g., a chess figure “flying” in the air) create yet another kind of object-scene consistency effect than objects at physically possible but unexpected locations (Võ and Wolfe, 2013). In addition, if we used four locations in the present study, at least two of the locations were in different quadrants of the image. This was done because previous work has shown that locations in different quadrants are sufficiently dissimilar from one another to exclude the possibility that each of the two locations would benefit from priming of the alternative location (Maljkovic and Nakayama, 1996). Accordingly, we also tried to locate a third and a fourth location in different quadrants wherever this was possible. Note also that weaker priming for the more distant of two alternative locations is even observed with distances between alternative locations much lower than this (Maljkovic and Nakayama, 1996). Such distances of a few grades of visual angle were realized in virtually all of the remaining cases. A between-participants manipulation of the number of locations was chosen to observe the maximal location priming effect (including also effects based on the participants' becoming aware of the repetition). Finally, for this manipulation, for each trial we either repeated the position of a certain object (this was the case in the single-location condition), or we pseudo-randomly drew one of two possible object locations (in the two-locations condition), or one of four possible object locations (in the four-locations condition). The constraint that we imposed on drawing and placing the objects was that each object was equally likely at each possible location—that is, each object was 50% likely at each of two positions in the two-positions condition, and each object was 25% likely at each of four positions in the four-positions condition. Consistent and inconsistent objects were presented equally likely at each position, and locations were to the left and the right sides of the images equally often. Examples of images used in Experiments 1 and 2 can be seen in Figure 1.

**Figure 2:**
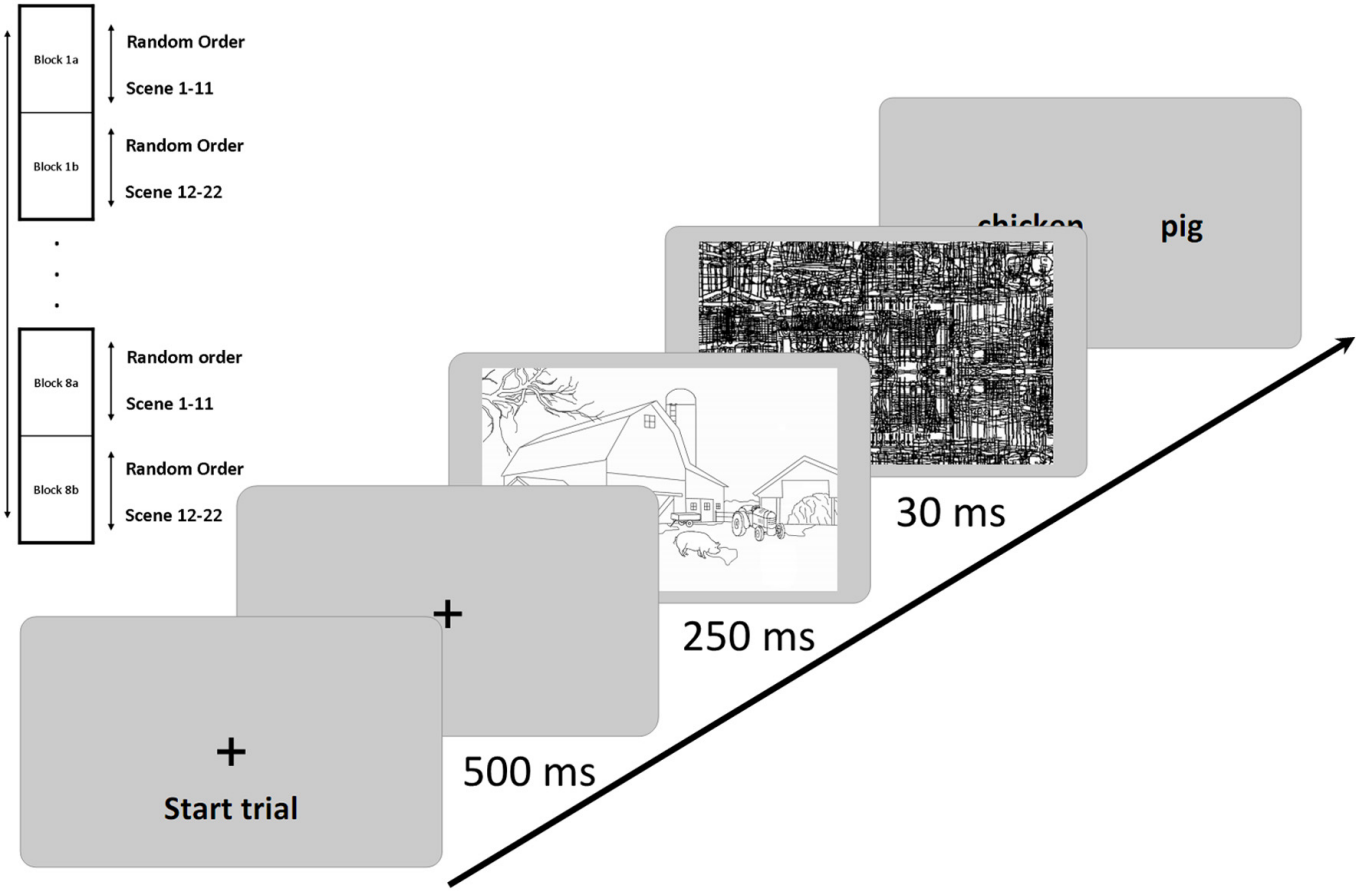
**Depicted is one trial of Experiments 1 and 2, and a scheme of how trials and blocks of trials were administered**. Each of eight blocks (1–8) contained all scenes (or backgrounds) and was divided into two sub-blocks (e.g., 1a and 1b) of 11 different scene and object images each, to avoid that one specific scene or object could be repeated in immediately succeeding trials. The arrow in the trial schema represents the direction of the flow of time; the sequence of events is from the lower left to the upper right.

Our procedure was adopted from Hollingworth and Henderson (1998) and, in the one-location condition the procedure was an exact replication of Experiment 4 of Hollingworth and Henderson (1998). One trial of the object recognition task proceeded as follows: once a cross centered on the screen disappeared (after 500 ms), a scene containing a consistent or an inconsistent object was presented for 250 ms, followed by a black and white noise-mask of 30 ms. Next, two words were shown, one left and one right, one denoting an object that was just presented, and the other one denoting an object that was not presented. To rule out a bias toward the report of consistent objects, the two words always both denoted either scene-consistent objects or inconsistent objects (Hollingworth and Henderson, 1998). Participants responded with a key on the side of the word denoting the presented object. Responses were given via the #*F* (left side) and #*J* keys (right side). Participants had to give fast and accurate responses via the #*F* key with the left index finger and the #*J* key with the right index finger. A schematic illustration of one trial of Experiments 1 and 2 is depicted in Figure 2.

After instructions and eight practice trials (with object-scene combinations not used during the experiment), eight blocks of 22 pictures each were randomly presented with the constraint of at least 11 trials between scene or object repetitions. The variables of interest were the two within-participant variables *consistency* (levels: consistent vs. inconsistent) and *scene repetitions* (levels: block 1 to block 8), and the between-participants variable *number of locations per scene* (levels: 1, 2, or 4 locations). For the latter, participants were subdivided into three equally-sized groups of 15 participants. Mean object eccentricities varied from 0 to 11.09° visual angle for the single-location condition (*M* = 6.10°), and from 0 to 13.10° visual angle for the multiple-locations conditions (two locations: *M* = 7.20°; four locations: *M* = 7.37°). Participants were instructed to look straight ahead at the center of the image during the whole presentation of the scene.

After the object recognition task, each participant rated the consistency of all 88 object-scene combinations. For the ratings, participants used the number keys without time pressure. In turn, each scene was shown separately with each of its possible target objects depicted below the scene. A scale ranging from #*1 (very fitting)* to #*5 (very non-fitting)* was shown at the bottom of the screen and participants rated how well each object fitted to the scene. The entire procedure lasted about 35 min.

### Results

For the following analyses of variance (ANOVAs) in all three experiments, we tested the critical pre-conditions of these ANOVAs by conducting Mauchly tests of the sphericity assumption. Where the sphericity assumption was violated, we reported the Greenhouse Geisser correction criterion epsilon and the corrected alpha level of the significance tests. All *post-hoc* tests are Bonferroni corrected. Results of the ANOVAs of recognition rates were cross validated with arc-sine transformed recognition rates, with mean RTs, and where possible with mean RTs of only the correct responses. The complementary analyses had different purposes. The ANOVA of the arc-sine transformed error rates compensated for the known correlation of the variance with the mean of the error rates (Winer et al., 1971) but it has the drawback of sometimes leading to false conclusions in the case of interactions (Jaeger, 2008). Therefore, it is good to confirm the results of each of these analyses by the corresponding alternative analysis. The ANOVA of all RTs ensured a picture of the performance during correct and incorrect object reports, and the ANOVA of only the correct RTs has the advantage of ensuring that the conclusions are based on the correct reports only. Again, it is reassuring if it can be shown that one of these analyses confirms the results of the respective alternative analysis. Results of these complementary analyses are only reported if deviating results were found.

Out of a total of 352 object-scene-location combinations, 10 object-scene combinations (or 40 object-scene-location combinations) were removed because at least one of the consistent objects was judged to be inconsistent or because one of the inconsistent objects was judged to be consistent based on the mean ratings of all subjects^5^. Another 11 out of the remaining 312 object-scene-location combinations were removed because the response rates exceeded the mean of all images by more than 2 *SD*s. Additionally, we discarded 3.8% data points with RTs exceeding subjects' individual mean RT by more than 2 *SD*s.

#### Analysis of location priming and scene repetition effects

A mixed-measurement ANOVA of recognition rates, with the two repeated-measures variables *consistency* (consistent vs. inconsistent) and *blocks/scene repetitions* (1–8), and the between-participants variable *number of locations per scene* (1, 2, or 4), led to main effects of consistency, *F*_(1, 42)_ = 8.93, *p* < 0.01, η^2^_p_ = 0.175, blocks/scene repetitions, *F*_(7, 294)_ = 7.63, *p* < 0.01, η^2^_p_ = 0.456, and number of object locations, *F*_(2, 42)_ = 12.04, *p* < 0.01, η^2^_p_ = 0.364. No significant interactions were found, all *F*s < 1.25, *p*s > 0.25.

Recognition rate was higher in consistent (*M* = 71%, *SD* = 17) than in inconsistent conditions (*M* = 69%, *SD* = 17), and steadily increased over blocks/scene repetitions with one exception (see Supplementary Material, Table A.2). The blocks/scene repetition effect reflected that performance in the first block differed significantly from the final three blocks, and that performance in the second block differed significantly from the 6th and the final blocks, all *p*s < 0.05. Recognition rate decreased from one location (*M* = 76%, *SD* = 17) over two (*M* = 68%, *SD* = 16) to four locations per scene (*M* = 66%, *SD* = 18), with significantly better performance with one location per object than with two and four locations per object, both *p*s < 0.01.

A corresponding mixed-design ANOVA of the mean RTs led to significant main effects of consistency, *F*_(1, 42)_ = 46.55, *p* < 0.01, and blocks/scene repetitions, *F*_(7, 294)_ = 74.67, *p* < 0.01. The main effect of number of object locations, *F* < 1.00, and all interactions, *F*s < 2.10 (*F*s < 2.70), were non-significant. RTs were lower in consistent (*M* = 1681 ms, *SD* = 543) than under inconsistent conditions (*M* = 1818 ms, *SD* = 606) and, with one exception, RTs decreased as a function of blocks/scene repetitions (see Table A.2). RTs significantly differed between all blocks, all *p*s < 0.05, except for a cluster consisting of the 3rd, 4th, and 5th block, and for a second cluster consisting of the 6th, 7th, and 8th block, which did not significantly differ from one another. For detailed statistics of the reported effects see Table A.2 in the Supplementary Material.

#### Analysis of eccentricity effects

Because mean eccentricity of objects was higher with several possible locations compared to one location per scene, we collapsed data across the variable locations per scene for ANOVAs with the variable *eccentricity* (low eccentricity < 6.5° visual angle vs. high eccentricity > 6.5° visual angle)^6^. One participant with lacking data in one cell was omitted from this analysis. For recognition rates, an ANOVA replicated significant effects of consistency, *F*_(1, 43)_ = 9.72, *p* < 0.01, η^2^_p_ = 0.184, and blocks/scene repetitions, *F*_(7, 301)_ = 7.69, *p* < 0.01, η^2^_p_ = 0.152. It also revealed a significant eccentricity effect, *F*_(1, 43)_ = 120.72, *p* < 0.01, η^2^_p_ = 0.737, and a significant interaction of Consistency × Eccentricity, *F*_(1, 43)_ = 5.68, *p* < 0.05, η^2^_p_ = 0.117, as well as a significant three-way interaction of Consistency × Eccentricity × Blocks/Scene Repetitions, *F*_(7, 301)_ = 2.22, *p* < 0.05, η^2^_p_ = 0.049. Consistency (consistent: *M* = 71%, *SD* = 22; inconsistent: *M* = 69%, *SD* = 26) and blocks/scene repetitions showed roughly the same effects as above (see Table A.3).

Performance was better for low (*M* = 76%, *SD* = 22) than for high eccentricities (*M* = 64%, *SD* = 25). Because mean eccentricity was higher with more variable object positions (2 or 4) than with a single object position, this eccentricity effect mimics an effect of location priming. Moreover, a consistency effect was found with high (consistent: *M* = 67%, *SD* = 23; inconsistent: *M* = 61%, *SD* = 26) but not with low object eccentricities (consistent: *M* = 76%, *SD* = 21; inconsistent: *M* = 76%, *SD* = 23). This interaction tended to develop over blocks/scene repetitions. Specifically, only in the 2nd, 6th, and 8th block, we found scene consistency effects for the more eccentric objects, all *t*s_(43)_ > 2.50, all *p*s < 0.05. No significant object-scene consistency effects were observed for objects of lower eccentricity, all *t*s_(43)_ < 1.30, all *p*s > 0.21.

A corresponding ANOVA of mean RTs revealed significant effects of consistency, *F*_(1, 43)_ = 32.44, *p* < 0.01, η^2^_p_ = 0.430, of blocks/scene repetitions, *F*_(7, 301)_ = 76.92, *p* < 0.01, η^2^_p_ = 0.641, and of eccentricity, *F*_(1, 43)_ = 132.79, *p* < 0.01, η^2^_p_ = 0.755. RT was lower in consistent (*M* = 1680 ms, *SD* = 610) than inconsistent conditions (*M* = 1807 ms, *SD* = 711), and decreased with block/scene repetitions (see Table A.3). RTs were also lower for less eccentric objects (*M* = 1567 ms, *SD* = 571) than for more peripheral objects (*M* = 1921 ms, *SD* = 705). The interactions of Consistency × Eccentricity, *F*_(1, 43)_ = 2.78, *p* = 0.10, and of Consistency × Eccentricity × Blocks/Scene Repetitions, *F*_(7, 301)_ = 1.29, *p* = 0.26, failed to become significant. For detailed statistics of the reported effects see Table A.3.

### Discussion

In the present experiment, we found an effect of scenes on object recognition—that is, an object-scene consistency effect: participants recognized scene-consistent objects better than inconsistent ones (Biederman et al., 1982). We also found that object recognition was better in later than earlier blocks, an effect that could be due to scene priming (Xu et al., 2007). Both effects were reflected in accuracy and RTs. Object-location priming (Maljkovic and Nakayama, 1996) was not certain. Recognition was better with more location priming/contextual cueing (i.e., only a single location) than with less location priming (i.e., if locations varied). However, eccentricity differences might equally account for this effect. A significant influence of location priming on the consistency effect was not found. The non-existent influences of location priming on consistency effects might suggest that visual search was not responsible for the consistency effects in the present study. For example, consistency effects might depend on the semantic relation between object and background (Bar, 2004, 2009). In line with this interpretation, consistency effects can also be found where visual search is ruled out because the objects are presented in the line of sight (Davenport and Potter, 2004). According to this view, priming during visual search and object-scene consistency effects could well be complimentary but independent principles of constraining visual processing in scenes to the most salient areas (Castelhano and Henderson, 2007; Malcolm and Henderson, 2010).

However, there is a caveat to this argument. Within the images, participants had to search for as many potential objects as possible because the participants were not informed about the particular objects of their judgments in advance of the images. (The words denoting the potential objects were only presented after the images.) Although many typical characteristics of visual search, such as serial vs. parallel search strategies, are aggravated under multiple-target search conditions (Thornton and Gilden, 2001, 2007), it is not so clear whether an influence of more- or less-expected object locations would also be augmented during search for multiple potential objects. Yet, of further interest regarding potential contributions of visual search to object-scene consistency effects, despite the lacking interaction between scene repetitions and consistency, we found a different indication that position knowledge might be responsible for the consistency effect: we found that eccentricity significantly interacted with consistency. Specifically, consistency affected the recognition of highly eccentric objects but not that of moderately eccentric objects. This finding aligns relatively well with the assumption that position knowledge could at least partly account for the consistency effect (Torralba et al., 2006): because response times were faster with objects closer to fixation than with objects presented away from fixation, consistency effects of the eccentric objects could have reflected more scene-specific use of prior knowledge about likely object locations. Following this argument, consistency effects based on strategic usage of position knowledge can only be observed when object perception becomes more difficult and knowledge about possible locations becomes more valuable. Additionally, object perception that relies on high special frequencies might take longer if the object is presented in the periphery. For this reason, too, participants might have had more time for scene processing before object identification with objects presented further in the periphery than with objects closer to fixation.

Also, in line with a boosting effect of scene priming on the consistency effect, the consistency effect tended to be only present with objects at peripheral locations and during the later blocks of the trials, when the scenes had been repeated several times.

One further result deserves a brief discussion. It is worth noting that we currently found a consistency effect where prior work did not find one—that is, in our conditions with a single location per object (Hollingworth and Henderson, 1998). The single-location condition was an exact replication of Hollingworth and Henderson. One important difference between the present study and that of Hollingworth and Henderson is that we only included object-scene pairings that the participants rated as consistent or as inconsistent as intended. Thus, we removed images that otherwise would have counteracted an object-scene consistency effect, whereas in the former study of Hollingworth and Henderson no such cleaning was reported. In fact, we found no significant difference between consistent (*M =* 65%) and inconsistent conditions (*M* = 68%), *t*_(14)_ < 1.00, when we used the unfiltered data and tested only the group without position changes, as it was done in the study of Hollingworth and Henderson.

## Experiment 2

Because Experiment 1 raised the question of whether eccentricity might have influenced object-scene consistency effects, Experiment 2 was conducted. In Experiment 2, we equated different levels of the variable number of locations per scene in terms of the average eccentricity of the objects. To that end, the pictures used in different locations were rearranged. As a consequence, we realized a comparable mean eccentricity of objects for all three levels of the variable locations per scene. Additionally, we controlled whether participants really held fixation (by eye tracking). By this means, we ensured that we were able to only use data in which the distance between fixation (at screen center) and object within the image was as intended.

### Methods

#### Participants

Thirty-six students (mean age 23 years) participated in exchange for course credit. All participants had normal or corrected-to-normal vision, and reported to have understood the instructions and the meaning of the target-object labels. Participants were naïve with respect to the hypotheses under investigation.

#### Apparatus

The apparatus was similar to the preceding experiment with the following exceptions. A computer with a CRT color monitor (resolution 1024 × 768 pixels; refresh rate 120 Hz) was used. Eye movements were monitored using a table-mounted Eyelink 1000 eye-tracker (SR Research) with 1000 Hz sampling rate.

#### Stimuli and procedure

Stimuli and procedure remained the same as in Experiment 1, except for two differences. First, participants did not perform a rating task at the end of the experiment. Instead, object-scene images were selected on the basis of the ratings from Experiment 1. Second, pictures were shifted between the location groups so that objects' mean eccentricities (*M* = 7.0°) were equated for the levels of the variable number of locations per scene (1, 2, or 4 possible locations). Prior to the experiment, the eye-tracking system was calibrated for each participant. This took about 5 min per participant. An entire experimental session lasted about 40 min.

### Results

One participant was removed from the data set because performance was at chance level and the mean RT was extremely low (940 ms compared to an average of 1776 ms in the whole sample). For all RT analyses, RTs exceeding subjects individual mean RT by ± 2 *SD*s were removed, which corresponds to 3.46 % of the data.

#### Analysis of location priming and scene repetition effects

We conducted a mixed-design ANOVA of recognition rates, with the two repeated-measures variables *consistency* (consistent vs. inconsistent) and *blocks/scene repetitions* (1–8), and the between-participants variable *number of locations per scene* (1, 2, or 4). A significant main effect was observed for consistency, *F*_(1, 32)_ = 6.76, *p* < 0.05, η^2^_p_ = 0.174, and scene repetitions, *F*_(7, 26)_ = 6.24, *p* < 0.01, η^2^_p_ = 0.627. Recognition rate was higher in consistent (*M* = 71%, *SD* = 17) than in inconsistent conditions (*M* = 67%, *SD* = 16), and, with one exception, increased over blocks/scene repetitions (see Table A.4). Neither the variable number of locations per scene nor one of the interactions became significant (all *p*s > 0.28).

A corresponding ANOVA of the RTs led to significant main effects of consistency, *F*_(1, 32)_ = 53.43, *p* < 0.01, η^2^_p_ = 0.625, and blocks/scene repetitions, *F*_(7, 26)_ = 16.85, *p* < 0.01, η^2^_p_ = 0.819. RTs were lower in consistent (*M* = 1634 ms, *SD* = 546 ms) than inconsistent conditions (*M* = 1840 ms, *SD* = 647 ms), and decreased with scene repetitions (see Table A.4 in the Supplementary Material). The main effect of number of locations per scene, *F* < 1.00, and all interactions, *F*s < 1.88, were non-significant. (For detailed statistics of the reported effects see Table A.4 in the Supplementary Material).

#### Analysis of eccentricity effects

The major variables of interest, number of locations per scene and blocks/scene repetitions, showed no significant interaction with object-scene consistency. The influence of number of locations per scene was eliminated when eccentricities were equated in Experiment 2. Therefore, the interaction of eccentricity and consistency that was found in Experiment 1 supposedly relies on eccentricity and not on locations per scene. To test whether eccentricity indeed again interacted with consistency, we submitted the data of Experiment 2 to a second ANOVA. This time, we collapsed across levels of the variable number of locations per scene *and* blocks/scene repetitions, and split conditions by their objects' eccentricities into two levels of low eccentricity vs. high eccentricity of objects^5^.

To control for the intended eccentricity in each trial, we included only those trials in which participants held their fixation during scene presentation. Two subjects had to be omitted from this analysis because they had no entries in at least one of the cells of the design. Of the remaining trials, a minimum of 57% and a maximum of 68% were retained for these analyses.

The corresponding analysis of the recognition rates (with variables consistency and eccentricity as in Experiment 1) confirmed one major result of Experiment 1. Significant main effects were found for consistency, *F*_(1, 32)_ = 4.94, *p* < 0.05, η^2^_p_ = 0.149, and for eccentricity, *F*_(1, 32)_ = 56.89, *p* < 0.01, η^2^_p_ = 0.634. No significant interaction could be observed for these factors, *F*_(1, 32)_ = 2.49, *p* = 0.12. Again, consistent objects (*M* = 72%, *SD* = 17) were recognized with a higher probability than inconsistent ones (*M* = 67%, *SD* = 17), and objects with low eccentricity (*M* = 77%, *SD* = 15) were reported more accurately than those with high eccentricity (*M* = 61%, *SD* = 14).

Moreover, we found significant main effects on RTs for the variables consistency, *F*_(1, 32)_ = 33.65, *p* < 0.01, η^2^_p_ = 0.513, and eccentricity, *F*_(1, 32)_ = 49.25, *p* < 0.01, η^2^_p_ = 0.606, as well as a significant interaction between these two variables, *F*_(1, 32)_ = 5.04, *p* < 0.05, η^2^_p_ = 0.136. Consistent objects were recognized faster (*M* = 1565 ms, *SD* = 466 ms) than inconsistent objects (*M* = 1759 ms, *SD* = 587 ms), and objects in the central regions of the images (*M* = 1508 ms, *SD* = 441 ms) were recognized faster than objects in the periphery (*M* = 1816 ms, *SD* = 582 ms). The consistency effect was more pronounced for objects in the periphery (consistent: *M* = 1677 ms, *SD* = 490 ms, vs. inconsistent: *M* = 1955 ms, *SD* = 639 ms) than at central positions (consistent: *M* = 1452 ms, *SD* = 418 ms, vs. inconsistent: *M* = 1564 ms, *SD* = 462 ms). For detailed statistics of the reported effects see Table A.5 in the Supplementary Material. (An analysis of only correct answers was impossible.)

### Discussion

**Figure 3:**
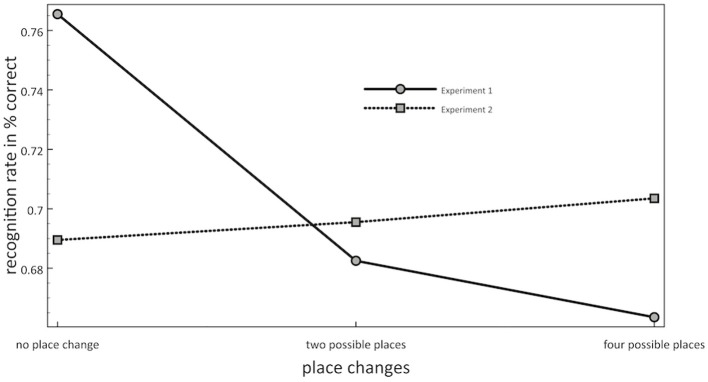
**Mean recognition rates (in percent correct) as a function of locations per object (from left to right: 1 location, 2 possible locations, 4 possible locations) and of Experiment (1: solid line; 2: dotted line)**.

The results of Experiment 2 confirmed the main findings of Experiment 1. As in Experiment 1, we observed a significant effect of scene context on object recognition. Consistent objects that were recognized both more accurately and faster than inconsistent objects. Also repetition priming of scenes was again found to be significant in Experiment 2. By contrast, the influence of the second priming effect (location priming/contextual cueing) could not be replicated in Experiment 2. Experiment 2 demonstrated that after controlling for object eccentricity, no effect of contextual cueing could be found for objects in outline sketches of natural scenes (see Figure 3).

Moreover, neither scene priming nor contextual cueing interacted with the consistency effect. With respect to scene priming, this influence should have boosted the consistency effect. With respect to contextual cueing, this influence should have led to a decrement of the consistency effect. Thus, both of these effects should have led to significant interactions but these interactions were not found.

## General discussion

The current study was concerned with two priming influences on object-scene consistency effects—that of contextual cueing and that of scene priming. When we manipulated the number of locations where each object could be presented, we found little evidence for contextual cueing. True, conditions with more contextual cueing (i.e., more object location repetitions) led to better performance than conditions with less location priming (i.e., less object location repetitions; Experiment 1). In addition, the object-scene consistency effect was lower in conditions with more contextual cueing than in conditions with less location priming. This pattern was predicted on the basis of the contextual-cueing account because contextual cueing should have been especially helpful to compensate for the difficult object recognition in inconsistent conditions but less so in consistent conditions. In the consistent conditions, object recognition should have been simply better from the outset of the experiment because of the average higher associations between objects and scenes in LTM.

**Figure 4:**
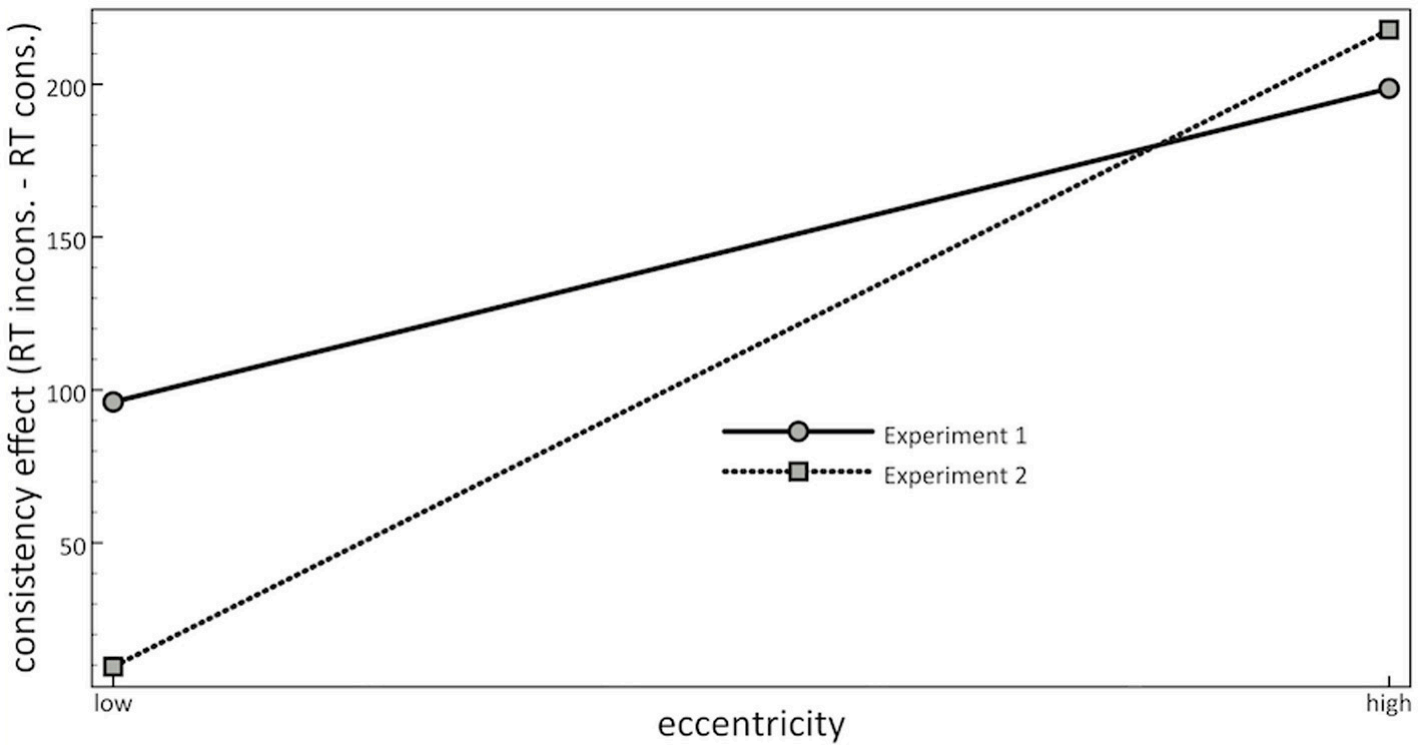
**Mean consistency effects in Reaction Times (RTs), calculated as RT of responses to inconsistent objects minus RT of responses to consistent objects, as a function of eccentricity (from left to right: low or high), and of Experiment (1: solid line; 2: dotted line)**. A positive value reflects faster reactions to consistent than inconsistent objects.

On closer scrutiny, however, it turned out that conditions with less contextual cueing were also of a higher average object eccentricity than conditions with more contextual cueing (Experiment 1). In addition, when different degrees of contextual cueing were equated for the mean object eccentricities, the influence of contextual cueing was non-significant (Experiment 2). Together, these results suggested that eccentricity provided a more powerful modulator of consistency effects than contextual cueing (see Figure 4).

Therefore, it could be that our manipulation of contextual cueing was simply too weak. In line with this speculation, we found little indications of a contextual cueing effect in a second respect. Contextual cueing could have shown up as a two-way interaction between (1) scene repetitions and (2) locations repetitions because the more scene repetitions have been seen by participants, the more contextual cueing should have taken effect. Yet, there was no such two-way interaction. Thus, we have an additional measure that confirms that contextual cueing did barely affect performance in the present study.

This leaves us with a puzzle. If eccentricity modulated the consistency effect, this modulation seems to be inconsistent with the fact that Davenport and Potter (2004) found a consistency effect even when presenting their targets at fixation. However, it is entirely possible that under the conditions of the present study, the semantic influence on object-scene consistency effect was lower and the effect of visual search on the consistency effect higher. Davenport and Potter presented their objects for only 80 ms and asked their participants to name the objects. Under these conditions, object-identification is probably more difficult than under the present study's conditions in which objects were shown for 250 ms and a two-alternative forced-choice discrimination was required. These differences in object identification difficulty could have been the decisive factor for whether one finds more eccentricity-independent consistency effects (mediated via, e.g., semantic associations) acting on a fast time scale or whether one finds more eccentricity-dependent consistency effects (mediated via, e.g., visual search) taking effect on a slightly longer time scale.

The second potential priming influence, that of scene priming, was also not certain. Importantly, this priming effect could have been reflected in the facilitated performance with increasing repetitions of each particular scene. However, in our first analysis, scene priming failed to interact with consistency although we would have expected a stronger object-scene consistency effect with more scene repetitions than with fewer scene repetitions because with each scene repetition, the speed of scene recognition and performance should have increased. Hence, we expected that the object-scene consistency effect that reflected the influence of scene recognition on object recognition would have increased with scene repetitions. With more scene priming and faster scene recognition, the differential impact of the scene of (1) slowing the recognition of inconsistent objects and (2) speeding up the recognition of consistent objects should have increased. However, besides an overall facilitation of performance, scene priming was without effect. In particular, the number of repetitions per scene image did not interact with the consistency effect.

Unexpectedly though, scene priming had an influence on the consistency effect in the form of a three-way interaction between eccentricity, scene repetitions, and consistency in Experiment 1^7^. This influence was in line with a boosting impact of scene priming on consistency effects because the interaction reflected that consistency effects were only significant when two conditions were met: (1) the objects were located in the periphery of the scene and (2) the participants had already seen a particular scene image a number of times. Clearly, however, we did not expect this particular three-way interaction and therefore caution is advised so as not to over-interpret this finding. This finding might reflect several principles besides scene priming. For example, it could also reflect more unspecific training effects because the number of scene repetitions was confounded with the time on task. In addition, because the three-way interaction was not replicated in the second experiment, it is possible that this interaction is too spurious to deserve an explanation.

### Further implications of the present findings

One also has to be careful not to overstate other implications of the present findings. First, one might be tempted to argue that the lack of priming effects is at odds with the assumption that visual search contributed to the consistency effect. As should be already clear from our discussion of the unexpected interaction between the eccentricity of the objects and the consistency effect, we think that visual search could have contributed to the consistency effect. In addition, a few other factors could have weakened the influences of visual search under the conditions of the present study. To note, we measured the consistency effect only with B/W outline sketches. These images were relatively unrealistic in the first place. It could well be that visual search behavior for more natural objects in more natural scenes relies more on LTM location knowledge, and that this particular search strategy was simply discouraged with the current experimental protocol. For instance, no color information and most of the texture information was unavailable in the outline sketches that we used. Even shape information was heavily simplified in the outline sketches. It could be that the participants therefore also abstained from relying on any LTM location information about possible target positions during their visual search. For these reasons, participants in the current study might have searched for the objects but not by any location-specific strategy. It would therefore be interesting to study the question of location priming in future studies with the help of photorealistic scene and object images.

Secondly, the consistency effect was observed under conditions in which Hollingworth and Henderson (1998) found no consistency effect. Part of the reason for the difference in the findings was that we measured the consistency effect with only that subset of the object-scene pairings that our participants also judged to be consistent or inconsistent, as it was intended. To note, not all object-scene pairings that were intended to be consistent were also judged to be consistent, and not all object-scene pairings that were intended to be inconsistent were also judged to be inconsistent. Whereas Hollingworth and Henderson did not check for such judgments by their participants and accordingly did not select the pictures on the basis of these subjective judgments, this selection was made in the current study. Also, in a further test, this selection turned out to be crucial. The consistency effect was absent [consistent: 65% vs. inconsistent: 68%; *t*_(14)_ < 1.00], when we tested in data (i.e., with all images, regardless of the participants' consistency judgments) and in conditions (i.e., the one location condition of Experiment 1) corresponding to an exact replication of Hollingworth and Henderson's Experiment 4.

Finally, all in all, however, the current findings could be also due to a residual consistency effect that cannot be attributed to visual search. This conclusion would be based on the fact that variables, such as contextual cueing that otherwise are diagnostic of visual search did not interact with the consistency effect. Our findings would thus be in line with other studies showing consistency effects where visual search was ruled out. For instance, Davenport and Potter (2004) showed a substantial consistency effect with objects presented in the line of sight. Under these conditions, participants did not have to search for the objects and yet a consistency effect was observed. Such findings indicate that more than visual search contributes to the consistency effect. For example, scene gist could be picked up during a fast processing phase based on low-spatial frequency (LSF) content. This gist could be used to select candidate object templates fitting to the scene context and based on LSF contour information of the objects (Bar, 2004, 2009). Furthermore, in the case of a consistent scene, these scene-informed *proto-objects* would then more often be immediately confirmed by a subsequent slower high-spatial frequency (HSF) processing phase, in which surface information about the objects becomes available. In contrast, in the case of an inconsistent scene, the HSF information would be more often disconfirming the gist-base selected proto-objects, thereby delaying object recognition (Bar, 2004, 2009). Due to the stimulation material in Experiment 1 and 2 these latter named principles might not have acted very strongly, explaining the comparably weak object-scene consistency effect. Future studies should therefore aim to test object-scene consistency effects with photorealistic scenes and objects.

## Author note

Nils Heise, Institut für Psychologische Grundlagenforschung, Universität Wien, Austria. This research was supported by a grant from the Wiener Wissenschafts-, Forschungs- und Technologiefonds (WWTF, Vienna Science and Technology Fund), grant no. CS 11-009 to Ulrich Ansorge, Shelley Buchinger, and Otmar Scherzer.

### Conflict of interest statement

The authors declare that the research was conducted in the absence of any commercial or financial relationships that could be construed as a potential conflict of interest.

## Footnotes

^1^The term visual search has been put into quotation marks because in a typical object-scene consistency experiment, the participants do not know which object will be probed. Thus, different from standard visual search experiments, participants have to search for and encode as many different objects as possible. Also different from standard visual search experiments, object-scene consistency effects are typically measured with very short display durations. To note, however, even with durations of less than 200 ms, visual search is possible under relatively demanding conditions (e.g., Dosher et al., 2010).

^2^Location priming can be found independently of the repetition of the context—that is, finding an object benefits from its repeated presentation at the same location even where the context changes (Maljkovic and Nakayama, 1996). By contrast, contextual cueing requires the repetition of the visual context alongside with that of the object's location for a facilitation of object search. Contextual cueing is the better fitting term in the current experiments because scenes were repeated together with the locations.

^3^Objects are part of a scene, and, hence, a scene repetition also leads to the repetition of the objects within a scene. Thus, one could argue that scene priming and object priming cannot be distinguished from one another. However, in an object-scene consistency study, such as the present one, it is more precise to distinguish between scene repetitions and object repetitions because only a quarter of each scene's repetitions contained one particular object of interest.

^4^Objects sizes were the same even if presented at slightly different virtual depth planes to rule out low-level feature differences (as spatial contrast) between conditions. (We considered the subtle depth-plane differences the lesser problem).

^5^We deleted all scenes rather than only particular object-scene combinations because we at least wanted to balance object and scene identities across conditions. For example, if only one object in a scene was judged consistent although it was inconsistent, we could have kept the remaining consistent-object condition of the same scene. However, as a consequence, object and scene identities in consistent vs. inconsistent scenes would not have been balanced across participants anymore.

^6^The mean eccentricity in Experiment 1 across all conditions was 244 pixels (6.5° visual angle). We mean-split conditions by the eccentricity of the objects rather than used eccentricity as a continuous variable because the mean-split allowed us to enter eccentricity as a two-level variable and to keep object identities balanced across different eccentricity conditions. (A more fine-grained correlation analysis would have confounded eccentricity and object influences because not all objects were placed in the same locations).

^7^Because of too many empty cells, blocks/scene repetitions could not be retained in an analysis of the eccentricity effects of only those trials in which fixation was held in Experiment 2.
