# Supplementary material for: The roles of scene priming and location priming in object-scene consistency effects
Source: Front Psychol. 2014 May 30;5:520. doi: 10.3389/fpsyg.2014.00520 (PMC4039012; doi:10.3389/fpsyg.2014.00520)
Supplement: Supplementary file 1 [file DataSheet1.DOCX]

## APPENDIX

## Tables

| TABLE A.1  *Full list of all object-scene combinations used in Experiments 1and 2. CO1 and CO2 denote the first and the second consistent object that was used, IO1 and IO2 denote the first and the second inconsistent object.* | | | | | | | | |
| --- | --- | --- | --- | --- | --- | --- | --- | --- |
| Scene |  | Objects | | | | | | |
|  |  | CO1 |  | CO2 |  | IO1 |  | IO2 |
|  | | | | | | | | |
| backyard |  | grill |  | dog |  | hallstand |  | pc |
| bar |  | wine bottle |  | cocktail glass |  | teddy |  | boots |
| bath |  | dryer |  | curler |  | sellotape |  | stapler |
| beach |  | swimming goggles |  | flippers |  | iron |  | hanger |
| bedroom |  | boots |  | teddy |  | wine bottle |  | cocktail glass |
| classroom |  | globe |  | backpack |  | wheelbarrow |  | mower |
| dining room |  | lamp |  | candle |  | tricycle |  | skateboard |
| pond |  | boot |  | bridge |  | pickup |  | bicycle |
| farm |  | chicken |  | pig |  | food processor |  | coffeemaker |
| front garden |  | wheelbarrow |  | mower |  | globe |  | backpack |
| gas station |  | pickup |  | bicycle |  | boat |  | bridge |
| kitchen |  | food processor |  | coffeemaker |  | chicken |  | pig |
| laundry |  | iron |  | hanger |  | swimming goggles |  | flippers |
| library |  | hallstand |  | pc |  | grill |  | dog |
| living room |  | armchair |  | television |  | hydrant |  | parking meter |
| locker room |  | dumbbell |  | sports shoes |  | flowers |  | jug |
| mall |  | shopping trolley |  | motorbike |  | cello |  | harp |
| office |  | stapler |  | sellotape |  | dryer |  | curler |
| playground |  | tricycle |  | skateboard |  | lamp |  | candle |
| restaurant |  | flowers |  | jug |  | dumbbell |  | sports shoes |
| street |  | hydrant |  | parking meter |  | armchair |  | television |
| theatre |  | cello |  | harp |  | shopping trolley |  | motorbike |
|  | | | | | | | | |

| TABLE A.2  *Descriptive statistics for Experiment 1: Means and Standard Deviations (SD) of Reaction Time (RT) and percentage correct answers (Acc) as a function of the experimental variables consistency, scene repetitions, and locations per scene .* | | | | | | | | | | |
| --- | --- | --- | --- | --- | --- | --- | --- | --- | --- | --- |
|  |  |  |  |  | | |  |  | | |
|  |  |  |  | Acc in % correct | | |  | RT in ms | | |
|  |  |  |  | Mean |  | SD |  | Mean |  | SD |
| Experiment 1: Consistency × scene repetitions × locations per scene | | | | | | | | | | |
| Consistency |  | Consistent |  | 71 |  | 17 |  | 1,681 |  | 543 |
|  |  | Inconsistent |  | 69 |  | 17 |  | 1,818 |  | 606 |
| Scene repetitions |  | Block 1 |  | 63 |  | 18 |  | 2,353 |  | 568 |
|  |  | Block 2 |  | 65 |  | 18 |  | 2,049 |  | 608 |
|  |  | Block 3 |  | 68 |  | 18 |  | 1,879 |  | 592 |
|  |  | Block 4 |  | 69 |  | 18 |  | 1,711 |  | 477 |
|  |  | Block 5 |  | 71 |  | 16 |  | 1,628 |  | 490 |
|  |  | Block 6 |  | 75 |  | 15 |  | 1,461 |  | 401 |
|  |  | Block 7 |  | 72 |  | 15 |  | 1,501 |  | 385 |
|  |  | Block 8 |  | 77 |  | 17 |  | 1,414 |  | 367 |
| Locations per scene |  | One location |  | 76 |  | 17 |  | 1,762 |  | 480 |
|  |  | Two locations |  | 68 |  | 16 |  | 1,712 |  | 637 |
|  |  | Four locations |  | 66 |  | 18 |  | 1,774 |  | 609 |
|  | | | | | | | | | | |

| TABLE A.3  *Descriptive statistics for Experiment 1: Means and Standard Deviations (SD) of Reaction Time (RT) and percentage correct answers (Acc) as a function of the experimental variables consistency, scene repetitions, and eccentricity.* | | | | | | | | | | |
| --- | --- | --- | --- | --- | --- | --- | --- | --- | --- | --- |
|  |  |  |  |  | | |  |  | | |
|  |  |  |  | Acc in % correct | | |  | RT in ms | | |
|  |  |  |  | Mean |  | SD |  | Mean |  | SD |
| Experiment 1: Consistency × scene repetitions × eccentricity | | | | | | | | | | |
| Consistency |  | Consistent |  | 72 |  | 22 |  | 1,680 |  | 610 |
|  |  | Inconsistent |  | 69 |  | 26 |  | 1,807 |  | 711 |
| Scene repetitions |  | Block 1 |  | 63 |  | 28 |  | 2,357 |  | 718 |
|  |  | Block 2 |  | 65 |  | 26 |  | 2,059 |  | 721 |
|  |  | Block 3 |  | 68 |  | 24 |  | 1,873 |  | 656 |
|  |  | Block 4 |  | 71 |  | 23 |  | 1,686 |  | 559 |
|  |  | Block 5 |  | 71 |  | 23 |  | 1,605 |  | 604 |
|  |  | Block 6 |  | 74 |  | 21 |  | 1,458 |  | 470 |
|  |  | Block 7 |  | 73 |  | 23 |  | 1,498 |  | 468 |
|  |  | Block 8 |  | 76 |  | 22 |  | 1,418 |  | 458 |
| Eccentricity |  | Low |  | 76 |  | 22 |  | 1,567 |  | 571 |
|  |  | High |  | 64 |  | 25 |  | 1,921 |  | 705 |
| Eccentricity | Low | Consistency | Consistent | 76 |  | 21 |  | 1,524 |  | 507 |
|  |  |  | Inconsistent | 76 |  | 23 |  | 1,610 |  | 627 |
|  | High |  | Consistent | 67 |  | 23 |  | 1,837 |  | 663 |
|  |  |  | Inconsistent | 61 |  | 26 |  | 2,005 |  | 736 |
|  | | | | | | | | | | |

| TABLE A.4  *Descriptive statistics for Experiment 2: Means and Standard Deviations (SD) of Reaction Time (RT) and percentage correct answers (Acc) as a function of the experimental factors consistency, scene repetitions and locations per scene .* | | | | | | | | | | |
| --- | --- | --- | --- | --- | --- | --- | --- | --- | --- | --- |
|  |  |  |  |  |  |  |  |  |  |  |
|  |  |  |  | Acc in % correct | | |  | RT in ms | | |
|  |  |  |  | Mean |  | SD |  | Mean |  | SD |
| Experiment 2: Consistency × scene repetitions × locations per scene | | | | | | | | | | |
| Consistency |  | Consistent |  | 71 |  | 17 |  | 1,634 |  | 546 |
|  |  | Inconsistent |  | 67 |  | 16 |  | 1,840 |  | 647 |
| Scene repetitions |  | Block 1 |  | 64 |  | 15 |  | 2,365 |  | 707 |
|  |  | Block 2 |  | 62 |  | 15 |  | 1,989 |  | 572 |
|  |  | Block 3 |  | 66 |  | 18 |  | 1,858 |  | 580 |
|  |  | Block 4 |  | 70 |  | 15 |  | 1,774 |  | 620 |
|  |  | Block 5 |  | 72 |  | 14 |  | 1,621 |  | 558 |
|  |  | Block 6 |  | 71 |  | 16 |  | 1,502 |  | 450 |
|  |  | Block 7 |  | 73 |  | 18 |  | 1,399 |  | 492 |
|  |  | Block 8 |  | 73 |  | 16 |  | 1,389 |  | 440 |
| Locations per scene |  | One location |  | 68 |  | 16 |  | 1,693 |  | 473 |
|  |  | Two locations |  | 69 |  | 16 |  | 1,763 |  | 687 |
|  |  | Four locations | | 70 |  | 17 |  | 1,751 |  | 894 |
|  | | | | | | | | | | |

| TABLE A.5  *Descriptive statistics for Experiment 2: Means and Standard Deviations (SD) of Reaction Time (RT) and percentage correct answers (Acc) as a function of the experimental variables consistency and eccentricity.* | | | | | | | | | | |
| --- | --- | --- | --- | --- | --- | --- | --- | --- | --- | --- |
|  |  |  |  |  |  |  |  |  |  |  |
|  |  |  |  | Acc in % correct | | |  | RT in ms | | |
|  |  |  |  | Mean |  | SD |  | Mean |  | SD |
| Consistency | Consistent |  |  | 72 |  | 17 |  | 1,565 |  | 466 |
|  | Inconsistent | |  | 67 |  | 17 |  | 1,759 |  | 587 |
| Eccentricity | Low |  |  | 77 |  | 15 |  | 1,508 |  | 441 |
|  | High |  |  | 61 |  | 14 |  | 1,816 |  | 582 |
| Eccentricity | Low | Consistency | Consistent | 83 |  | 18 |  | 1,452 |  | 418 |
|  |  |  | Inconsistent | 74 |  | 33 |  | 1,564 |  | 462 |
|  | High |  | Consistent | 62 |  | 27 |  | 1,677 |  | 490 |
|  |  |  | Inconsistent | 61 |  | 26 |  | 1,955 |  | 639 |
|  | | | | | | | | | | |
